# Measurement of Entrance Skin Dose and Calculation of Effective Dose for Common Diagnostic X-Ray Examinations in Kashan, Iran

**DOI:** 10.5539/gjhs.v7n5p202

**Published:** 2015-02-24

**Authors:** Akbar Aliasgharzadeh, Ehsan Mihandoost, Mahboubeh Masoumbeigi, Morteza Salimian, Mehran Mohseni

**Affiliations:** 1 Department of Radiology and Medical Physics, Faculty of Paramedical Sciences, Kashan University of Medical Sciences, Kashan, Iran; 2 Department of Medical laboratory, Faculty of Paramedical Sciences, Kashan University of Medical Sciences, Kashan, Iran

**Keywords:** entrance skin dose, effective dose, common diagnostic x-ray examination

## Abstract

The knowledge of the radiation dose received by the patient during the radiological examination is essential to prevent risks of exposures. The aim of this work is to study patient doses for common diagnostic radiographic examinations in hospitals affiliated to Kashan University of Medical sciences, Iran. The results of this survey are compared with those published by some national and international values. Entrance surface dose (ESD) was measured based on the exposure parameters used for the actual examination and effective dose (ED) was calculated by use of conversion coefficients calculated by Monte Carlo methods. The mean entrance surface dose and effective dose for examinations of the chest (PA, Lat), abdomen (AP), pelvis (AP), lumbar spine (AP, Lat) and skull (AP, Lat) are 0.37, 0.99, 2.01, 1.76, 2.18, 5.36, 1.39 and 1.01 mGy, and 0.04, 0.1, 0.28, 0,28, 0.23, 0.13, 0.01 and 0.01 mSv, respectively. The ESDs and EDs reported in this study, except for examinations of the chest, are generally lower than comparable reference dose values published in the literature. On the basis of the results obtained in this study can conclude that use of newer equipment and use of the proper radiological parameter can significantly reduce the absorbed dose. It is recommended that radiological parameter in chest examinations be revised.

## 1. Introduction

Nowadays human organ imaging is performed by different systems and methods. As the new diagnostic methods including CT, MRI, and sonography grow, radiography is to be in progress as well, because it is still a powerful tool with enough benefits for the patients undoubtedly. Therefore, patients’ exposure to radiation has been increased all over the world due to this diagnostic radiography (The 2007 Recommendations of the International Commission on Radiological Protection. ICRP publication 103, 2007; European Commission, European Guidance on Estimating Population Doses from Medical X-Ray Pocedures. Radiation Protection N.154, 2008; Fazel et al., 2009; Hart, Hillier, & Shrimpton, 2010; United Nations Scientific Committee Effects Atomic Radiation, 2010). On the other hand, in researches have done in various parts of the world can be seen large difference in patient dose within and between hospitals for the same examination and for the same average patient size (Abdelhalim, 2010; Kharita, Khedr, & Wannus, 2010; Osei & Darko, 2013; Shahbazi-Gahrouei & Baradaran-Ghahfarokhi, 2013; Shirin Shandiz, Bahreyni Toosi, Farsi, & Yaghobi, 2014; Sonawane, Shirva, & Pradhan, 2010; Zenone et al., 2012). Since using ionising X-rays is associated with some risk of developing cancer, the basic radiation protection concept or philosophy ALARA states that ’all exposures must always be keeping As Low As Reasonably Achievable (National Council on Radiation Protection and Measurements, 1990). So, the knowledge of the radiation dose received by the patient during the radiological examination is essential to prevent risks of exposures that involve a great number of people. Various indicators are used to estimate detriment from cancer and genetic effects of radiation. According to ICRP 60, the basic quantity associated with the risk of deleterious effects on health is the effective dose that is the valuable and central quantity for dose limitation in the field of radiological protection of the patient (International Commission on Radiological Protection, 1991). This dose descriptor is being increasingly used to determine the quantity of radiation dose received by patient undergoing diagnostic X- ray examinations (Brenner & Huda, 2008; Kharita et al., 2010; Mettler, Huda, Yoshizumi, & Mahesh, 2008; Osei & Darko, 2013; Shahbazi-Gahrouei & Baradaran-Ghahfarokhi, 2013; Teles et al., 2013). Whereas effective dose (ED) is affected by patient structure and radiological method; therefore, the calculation of this quantity is of utmost importance. Because it is almost impossible to directly measure effective dose during clinical procedures, it must be determined indirectly. In general, indirect estimates of effective dose starts from incident air kerma (K_a, i_) measurements as input parameters and uses dedicated conversion coefficients (European Commission, European Guidance on Estimating Population Doses from Medical X-Ray Pocedures. Radiation Protection N.154, 2008; International Atomic Energy Agency, 2007; International Commission Radiation Units, 2005). The purpose of this study was to measure the entrance surface dose (ESD) and estimate the ED for several common X-ray projections of adult patients. The study is the first of its kind to be carried out for five routine radiographies (8 projections) in hospitals affiliated to Kashan University of Medical sciences, Iran. The results of this survey were compared with those published by some national and international values.

## 2. Methods

This study included the eight most common performed diagnostic X-ray examinations, that is; Posterior Anterior (PA) and Lateral (Lat) chest, Anterior Posterior (AP) abdomen, AP pelvis, AP and Lat lumbar spine, and PA and Lat skull. This study was carried out in four university hospitals. The radiographic equipments used in this study were all analogue, the total filtration ranged from 2.00 to 3.5 mmAl and the film screen combination speed was 400. Radiographic factors included tube potential (kVp), exposure setting (mAs), and focus skin distance (FSD) that were normally used in each radiology room by radiographers for average size adult patients (with weights between 60–80 kg according to the European guideline) for only suitable diagnostic quality images as distinct by the radiologist (European Commission, European Guidance on Estimating Population Doses from Medical X-Ray Pocedures. Radiation Protection N, 154, 2008).

To calculate the ED, the K_a,i_ at the same FSD on the beam central axis was firstly measured with a flat chamber type 77334 connected to a radiation dosemeter UNIDOSE made by PTW company calibrated for measuring the air kerma in the range of energy between 40-150 kVp (calibrated by the secondary standard dosemetry laboratory in the Atomic Energy Organization of Iran). The ionisation chamber was placed on a material with a low scatter level (a flat cardboard).

Then, the amount of ESD was achieved from multiplying the K_a,i_ quantity in back scatter factor (BSF) coefficient. Finally conversion coefficients calculated by Monte Carlo methods were used to relate ESD to ED. To correctly apply these factors, it is required to know the tube potential and the field size used in clinical practice and total filtration of radiographic equipments (International Atomic Energy Agency, 2007; International Commission Radiation Units, 2005).

## 3. Results and Discussion

Mean values of kVp, mAs, and FSD, along with their range for each type of radiological examination obtained in the four hospitals are given in Table 1.

**Table 1:** Mean values of exposure parameters for five routine x-ray examinations (8 projections). Range are shown in parentheses

Examination	Hospital	Potential (kVp)	exposure setting (mAs)	focus skin distance (FSD)
Chest PA	Beheshti	70(63-76)	19(16-22)	156(151-168)
	Naghavi	69(60-79)	20(16-25)	117(78-129)
	Shbihkhani	72(63-77)	18(14-20)	114(108-128)
	Syidoshohada	58(52-65)	19(10-29)	120(115-128)

Chest Lat	Beheshti	81(75-96)	33(28-40)	147(136-156)
	Naghavi	75(66-85)	33(25-40)	115(105-123)
	Shbihkhani	81(75-86)	32(16-44)	102(98-105)
	Syidoshohada	66(64-69)	24(13-36)	114(110-120)

Abdomen	Beheshti	75(70-83)	24(13-36)	84(78-88)
	Naghavi	65(60-75)	23(20-25)	73(69-79)
	Shbihkhani	75(71-77)	25(20-29)	78(76-83)
	Syidoshohada	65(62-71)	26(16-32)	78(75-84)

Pelvis	Beheshti	72(65-75)	22(16-32)	88(79-97)
	Naghavi	63(59-70)	20(16-25)	72(69-78)
	Shbihkhani	76(75-80)	24(19-26)	78(68-85)
	Syidoshohada	65(58-71)	27(20-32)	80(76-84)

Lumbar Spine AP	Beheshti	75(68-79)	24(13-36)	86(80-95)
	Naghavi	69(63-75)	22(16-32)	75(64-78)
	Shbihkhani	75(66-85)	24(20-26)	80(75-84)
	Syidoshohada	71(64-85)	28(20-40)	77(72-81)

Lumbar Spine Lat	Beheshti	82(77-88)	40(23-63)	77(72-84)
	Naghavi	78(68-85)	34(32-40)	69(67-70)
	Shbihkhani	83(74-90)	43(28-58)	72(66-78)
	Syidoshohada	80(75-89)	44(32-64)	70(65-74)

Skull PA	Beheshti	68(64-70)	19(16-32)	83(80-87)
	Naghavi	62(60-67)	20(16-25)	73(72-79)
	Syidoshohada	61(58-68)	22(16-32)	84(83-87)

Skull Lat	Beheshti	65(62-70)	17(16-18)	87(85-90)
	Naghavi	59(55-65)	19(16-20)	86(83-86)
	Syidoshohada	57(54-64)	20(13-29)	87(84-90)

Table 2 shows the mean, standard error of mean of ESD (mGy) and the mean of ED (mSv) values of five radiographic examinations (8 projections) obtained in the four hospitals.

**Table 2:** Mean values of ESD (mGy) and the ED (mSv) for five routine x-ray examinations (8 projections). Standard error of means are shown in parentheses

Hospital	Beheshti		Naghavi		Shbihkhani		Syidoshohada	
			
Examination	ESD	ED	ESD	E.D	ESD	ED	ESD	ED
Chest PA	0.31(0.01)	0.03	0.40(0.02)	0.04	0.65(0.04)	0.07	0.43(0.07)	0.04
Chest Lat	0.88(0.04)	0.09	1.07(0.10)	0.11	1.56(0.19)	0.16	1.09(0.36)	0.11
Abdomen	1.62(0.06)	0.23	2.92(0.11)	0.41	2.46(0.34)	0.34	2.81(0.39)	0.39
Pelvis	1.21(0.05)	0.19	2.89(0.23)	0.46	2.70(0.31)	0.43	2.80(0.48)	0.45
lumbar Ap	1.50(0.06)	0.16	4.17(0.63)	0.45	2.45(0.30)	0.26	3.37(0.37)	0.36
lumbar Lat	4.10(0.20)	0.10	8.92(0.57)	0.22	6.51(1.21)	0.16	7.12(0.66)	0.18
Skull PA	0.98(0.04)	0.01	2.83(0.09)	0.03	**	**	1.84(0.14)	0.02
Skull Lat	0.80(0.03)	0.01	1.70(0.07)	0.02	**	**	1.36(0.12)	0.01

** Indicates data not available in some hospitals during time of study.

As it is seen in the tables 1 and 2, the lowest mean ESDs and EDs were observed at Beheshti hospital, while the highest mean ESDs and EDs were seen at Shbihkhani hospital for the chest PA and Lat projections. It seems that decreased dose level in the patients may be due to use of newer equipment and large FSD which is 156 and 147 cm in the former hospital compared to 117 and 115 cm in the latter hospital for the AP and Lat projections.

For the rest of the examination, the lowest mean ESDs and EDs were observed at Beheshti hospital, but the highest mean of these parameters were seen at Naghavi hospital because of older X-ray equipment and short FSD use in these hospital.

Table 3 compares the mean values of kVp and mAs obtained in this study with those in IRAN (Asadinezhad & Toossi, 2008), UK 2010 (Hart et al., 2010), and with the EC recommendations (European Commission, 1998).

**Table 3:** Comparison of mean radiographs exposure parameters values with other studies for common radiography for each type of radiological examination

Examination	This study		IRAN(20)		UK2010 (4)		EC

	kV	mAs	kV	mAs	kV	mAs	KV
Chest PA	69	19	66	18	88	5	125
Chest Lat	79	32	72	41	89	13	125
Abdomen	73	24	68	54	76	41	75-90
Pelvis	70	22	66	48	75	33	75-90
lumbar Ap	74	24	70	50	78	46	75-90
lumbar Lat	82	40	80	73	89	56	80-95
Skull PA	66	19	64	42	72	20	70-85
Skull Lat	63	18	59	32	66	11	70-85

Tables 4 and 5 compares the mean values of measured ESDs (mGy) and the calculated effective doses (mSv), for each of examinations in this study with corresponding values reported in the other studies; IRAN (Asadinezhad & Toossi, 2008), UK 2010 (Hart et al., 2010), UK (Wall et al., 2011), IAEA (Muhogora et al., 2008) and UNSCEAR 2008 (United Nations Scientific Committee Effects Atomic Radiation, 2010) reference doses.

**Table 4:** Comparison of measured mean ESD (mGy) values with other studies for common radiography

Examination	This study	IRAN(20)	UK2010(4)	IAEA(21)
Chest PA	0.37	0.41	0.15	0.33
Chest Lat	0.99	2.7	0.5	**
Abdomen	2.01	4.06	4	3.64
Pelvis	1.76	3.18	4	3.68
lumbar Ap	2.18	3.43	5.7	4.07
lumbar Lat	5.36	8.41	10	8.53
Skull PA	1.39	2.83	1.8	2.41
Skull Lat	1.01	1.93	1.1	**

Note.

** indicates data not available.

**Table 5:** Comparison of calculated mean EDs (mSv) values with other studies for common radiography

Examination	This study	UK E-60(22)	UK E-103(22)	UNSCEAR2008(5)
Chest PA	0.04	0.014	0.014	0.05
Chest Lat	0.10	0.031	0.038	0.2
Abdomen	0.28	0.47	0.43	0.8
Pelvis	0.28	0.45	0.28	1
lumbar Ap	0.23	0.41	0.39	1.2
lumbar Lat	0.13	0.26	0.21	1.2
Skull PA	0.01	0.016	0.020	**
Skull Lat	0.01	0.012	0.016	**

Note.

** indicates data not available.

Comparing the exposure parameters values, measured mean ESD and calculated ED values applied in this study with the guide levels of UK 2010 (Hart et al., 2010), UK (Wall et al., 2011), IAEA (Muhogora et al., 2008) references for chest PA and Lat projections reveals that ESD and ED are above the guide levels. The major reason for this overdose is lower potential values and the higher mAs values used in this study. However, ESD for chest PA projection is similar to IRAN (Asadinezhad & Toossi, 2008) because the same parameters were used in these studies. But for the rest of the examination, the ESD and ED values were lower than the values found by the IRAN (Asadinezhad & Toossi, 2008), UK 2010 (Hart et al., 2010), UK (Wall et al., 2011), IAEA (Muhogora et al., 2008) and UNSCEAR 2008 (United Nations Scientific Committee Effects Atomic Radiation, 2010) reference doses. The difference in the ESD and ED values may be due to differences in the exposure conditions that the potential values are more or less similar but mAs values in this study is about half of other studies.

## 4. Conclusion

On the basis of the results obtained in this study can conclude that use of newer equipment and use of the proper radiological parameter such as the large distance between patient and x-ray source, high tube potential and low tube current can significantly reduce the absorbed dose especially by the low cost means proper of radiological parameter.

Due to the universality and high percentage of chest X-rays requests and the important role of this test in patient’s cumulative doses, specific strategies must be performed to reduce patient dose in this test.
